# Draft Genome Sequence of *Anaerolineae* Strain TC1, a Novel Isolate from a Methanogenic Wastewater Treatment System

**DOI:** 10.1128/genomeA.01104-15

**Published:** 2015-10-08

**Authors:** Norihisa Matsuura, Dieter M. Tourlousse, Liwei Sun, Mayu Toyonaga, Kyohei Kuroda, Akiko Ohashi, Rodrigo Cruz, Takashi Yamaguchi, Yuji Sekiguchi

**Affiliations:** a Biomedical Research Institute, National Institute of Advanced Industrial Science and Technology (AIST), Tsukuba, Ibaraki, Japan; b School of Energy & Environment, Southeast University, Nanjing, Jiangsu, China; c Department of Civil and Environmental Engineering, Nagaoka University of Technology, Nagaoka, Niigata, Japan; d EPAS International NV, Ghent, Belgium

## Abstract

We report the draft genome sequence of *Anaerolineae* bacterium strain TC1, newly isolated from a methanogenic wastewater treatment system. The assembly contains 16 contigs in 3 scaffolds representing 3,510,630 bp in total with a G+C content of 41.35%. The genome is predicted to contain 2,793 protein-coding genes and 56 RNAs.

## GENOME ANNOUNCEMENT

Cultivation-independent surveys have demonstrated that members of the class *Anaerolineae* are frequent inhabitants of a wide range of ecosystems (1). However, the taxon is currently represented by only a few cultured isolates, limiting our understanding of the phenotypic traits harbored within the class *Anaerolineae*. To further explore the genomic and physiological diversity of this class, a novel *Anaerolineae* strain (designated strain TC1) was isolated from a methanogenic wastewater treatment system operated in Hungary. The 16S rRNA gene sequence of strain TC1 (DDBJ/EMBL/GenBank accession number LC049585) shared less than 90% similarity to those of other characterized *Anaerolineae* species, with *Ornatilinea apprima* strain P3M-1 (accession number JQ292916) showing the highest 16S rRNA gene sequence identity (88.3%) to strain TC1. This suggests that strain TC1 may represent a novel genus within the class of the *Anaerolineae*. We determined the genome sequence of strain TC1, to complement its ongoing physiological characterization.

The draft genome of *Anaerolineae* bacterium strain TC1 was produced by constructing indexed Nextera XT sequencing libraries with 300- to 700-bp insert sizes and Nextera mate-pair (2 to 10 kb) sequencing libraries for the genomic DNA extracted from the strain. Pooled paired-end and mate-pair libraries were used for sequencing using an Illumina MiSeq instrument to generate 2 × 250-bp paired-end reads with average coverage of 50×. Mate-pair libraries were also sequenced on the MiSeq with 2 × 250-bp paired-end reads to obtain 10× coverage of the genome. Illumina sequence raw reads were merged with SeqPrep (https://github.com/jstjohn/SeqPrep) using default settings, and Illumina sequencing adapters were removed. Unmerged reads were quality trimmed and filtered using Nesoni v0.112 (https://github.com/Victorian-Bioinformatics-Consortium/nesoni). The merged and trimmed reads were assembled using SPAdes v2.5.0 (2). Further scaffolding and manual refinement of the assembly using the mate-pair data were performed as described previously (3). Annotation of the genome was performed within the Integrated Microbial Genomes (IMG) platform (4).

The final high-quality draft assembly of *Anaerolineae* strain TC1 genome contains 16 contigs in 3 scaffolds; average genome coverage was 60×. The genome size (3,510,630) is comparable to that of other *Anaerolineae* species (3.69 to 4.44 Mbp) while the G+C content (41.35%) is lower (46.82% to 57.36% for other *Anaerolineae* species) (5). Annotation indicated the presence of 2,793 protein-coding and 56 RNA genes; two sets of rRNA genes were identified. The vast majority of *Chloroflexi*-specific phylogenetic marker genes (179 out of 198 genes, 90.4%) were identified using Phyla-AMPHORA (6), indicating that the draft genome is nearly complete. We expect that the availability of a draft genome of strain TC1, together with other *Anaerolineae* genomes (5), will shed light on the metabolic potential and possible ecological role of members of the class *Anaerolineae* in different ecosystems.

### Nucleotide sequence accession numbers.

The whole-genome shotgun project has been deposited at DDBJ/EMBL/GenBank under the accession number BBYH00000000 for *Anaerolineae* bacterium strain TC1. The version described in this paper is version BBYH01000000.
